# Renal Squamous Cell Carcinoma Presenting With Renohepatic Fistula: A Rare Amalgam

**DOI:** 10.31486/toj.22.0039

**Published:** 2023

**Authors:** Bharti Varshney, Aasma Nalwa, Taruna Yadav, Gautam Choudhary

**Affiliations:** ^1^ Department of Pathology, All India Institute of Medical Sciences, Jodhpur, Rajasthan, India; ^2^ Department of Diagnostic and Interventional Radiology, All India Institute of Medical Sciences, Jodhpur, Rajasthan, India; ^3^ Department of Urology, All India Institute of Medical Sciences, Jodhpur, Rajasthan, India

**Keywords:** *Carcinoma–squamous cell*, *kidney neoplasms*, *nephrolithiasis*, *pyelonephritis–xanthogranulomatous*

## Abstract

**Background:** Primary squamous cell carcinoma (SCC) of the kidney, a rare malignancy that accounts for less than 1% of all urinary tract malignancies, is usually diagnosed in late stages because of the lack of characteristic clinical and imaging features and aggressive behavior.

**Case Report:** A 66-year-old male presented with complaints of right flank pain. Imaging suggested the differential diagnoses of xanthogranulomatous pyelonephritis or renal malignancy extending into segment VI of the liver. Right subcapsular nephrectomy was performed, and nonbilious fluid from the liver cavitary lesions was drained. Histopathologic examination showed that the lesion was a renal SCC with contiguous malignant infiltration of the liver that led to a renohepatic fistula.

**Conclusion:** Renal SCC is a rare high-grade neoplasm and can present in an unusual form with a poor prognosis.

## INTRODUCTION

Primary squamous cell carcinoma (SCC) of the kidney, a rare malignancy that accounts for less than 1% of urinary tract malignancies,^1^ is usually associated with nephrolithiasis and hydronephrosis. Calculi in the kidney are a cause of chronic irritation causing squamous metaplasia that can become malignant.^2,3^ SCC of the kidney, in contrast to other renal tumors, is usually diagnosed late because of nonspecific clinical and radiologic findings. We present a case of kidney SCC that led to a renohepatic fistula diagnosed after nephrectomy for suspected xanthogranulomatous pyelonephritis.

## CASE REPORT

A 66-year-old male presented with complaints of right flank pain for 20 days that he described as mild to moderate in intensity; nonradiating; and not associated with fever, vomiting, hematuria, or pyuria. The patient had no history of smoking but did have a history of right-sided pyelolithotomy for renal calculi 35 years prior. On examination, he had right flank tenderness without any palpable mass. His leukocyte count was 15,800 cells/mm^3^ (reference range, 4,000-11,000 cells/mm^3^), predominantly neutrophilic. He had elevated C-reactive protein of 105.7 mg/L (reference range, 8-10 mg/L) and elevated erythrocyte sedimentation rate of 91 mm/h (reference range, 0-20 mm/h). The patient's kidney, liver function, and other biochemical tests were within normal limits. On the day of admission, he was started on a 3 times daily intravenous (IV) infusion of cefoperazone-sulbactam 1.5 g because of the suspicion of urinary tract infection.

**Figure 1.**
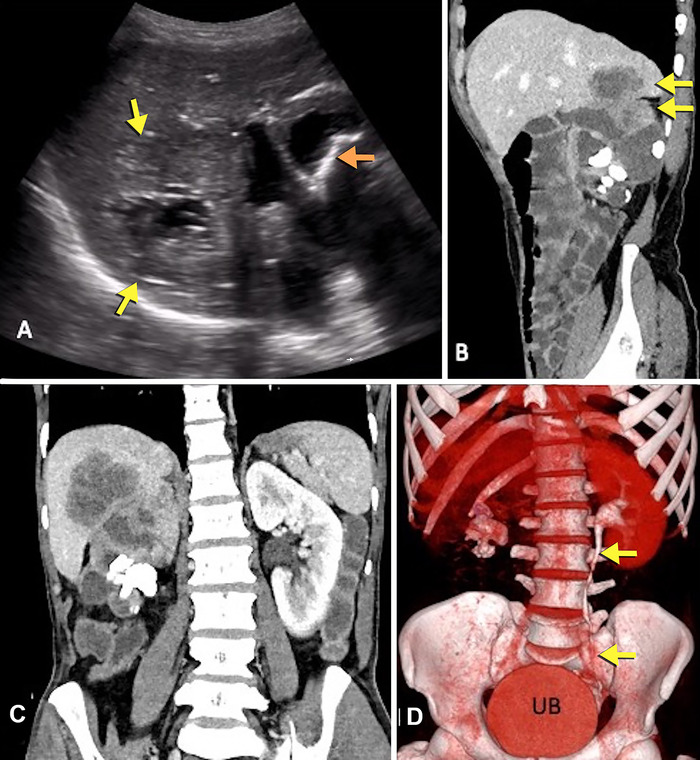
**(A) Sagittal ultrasound image shows a heterogeneous mass in the upper pole of the right kidney with contiguous extension in segment VI of the liver and a central necrotic component (arrows on the left). Renal calculus with distal acoustic shadowing (arrow on the right) and hydronephrosis are also present. (B) Sagittal and (C) coronal reformats of contrast-enhanced computed tomography show multiple right staghorn renal calculi with hydronephrosis. A heterogeneously enhancing mass in the upper pole of the right kidney infiltrates the adjacent liver (arrows). (D) Excretory phase volume-rendered image shows right staghorn calculus with nonfunctioning right kidney. The left renal pelvis, ureter (arrows), and urinary bladder are delineated due to normal contrast excretion.** UB, urinary bladder.

Abdominal ultrasound showed an ill-defined heterogeneous mass in the upper pole of the right kidney, extending into segment VI of the liver with no significant vascularity. The liver component showed central necrotic areas (Figure 1A). On further evaluation with contrast-enhanced computed tomography, a 3.5-cm staghorn calculus was visualized in the right renal pelvis with other small calculi in the pelvicalyceal system, leading to moderate hydronephrosis (Figures 1B and 1C). The right kidney was nonfunctioning with an absence of contrast excretion in the delayed phase (Figure 1D). Multiple necrotic lymph nodes were seen in the right renal hilar and retroperitoneal (para-aortic, aortocaval) region. The right renal vein and inferior vena cava showed normal contrast opacification with no evidence of thrombus. The left kidney was normal.

Three samples of the patient's urine were negative for malignant cytology, and culture was negative for the growth of any microorganisms. On day 4 of admission, the patient's total leukocyte count increased to 18,600 cells/mm^3^ despite IV antibiotics. Given the patient's nephrolithiasis, preoperative differential diagnoses were xanthogranulomatous pyelonephritis with an extension of infection to the liver or renal malignancy.

**Figure 2.**
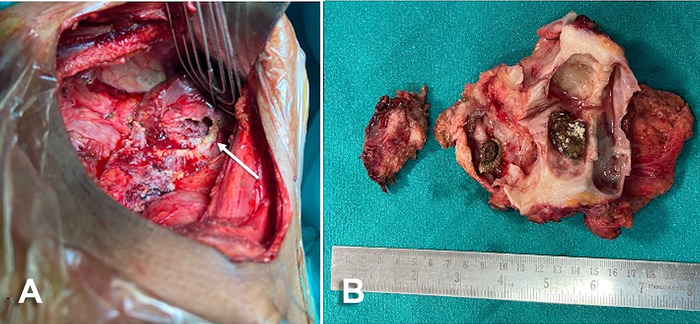
(A) Intraoperative image shows cavitary lesion in the inferior part of the liver (arrow). (B) Cut section of the kidney displays hydronephrosis, renal cortical atrophy, yellowish discoloration of pelvicalyceal lining, multiple calculi, thickening of intercalyceal septa, and renal pelvis.

The patient underwent a right open subcapsular nephrectomy through an extraperitoneal incision on day 6 of admission. The kidney was adherent to the parietal wall. The 2 cavitary lesions in the liver were filled with turbid nonbilious fluid and were drained (Figure 2A). The peritoneal cavity was thoroughly washed with saline, and a small peritoneal opening at the inferior margin of the liver was repaired. The postoperative period was uneventful, and the patient was discharged on postoperative day 6 with oral cefixime 200 mg twice daily for 5 days.

On gross examination of the nephrectomy specimen (Figure 2B), the kidney was mildly enlarged. The cut section of the kidney showed multiple variable-sized calculi. The wall of the kidney was diffusely thickened, and no definite intraluminal growth was identified.

**Figure 3.**
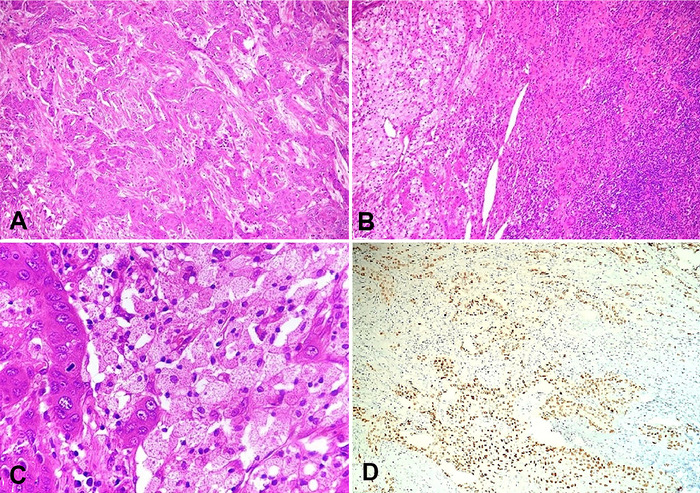
(A) Photomicrograph shows a tumor arranged in nests and islands with adjacent renal parenchyma in the lower left corner displaying atrophied interstitium and tubules (hematoxylin and eosin stain [H&E], magnification ×40). (B) Photomicrograph demonstrates the focal replacement of renal parenchyma with foamy histiocytes and lymphoplasmacytic inflammation (H&E, magnification ×100). (C) High-power view displays prominent histiocytes with the adjacent focus of the tumor (H&E, magnification ×400). (D) Photomicrograph shows tumor cells immunopositive for p40 (magnification ×100).

On microscopy (Figure 3), the renal parenchyma was infiltrated with nests and sheets of epithelial cells, polygonal in shape and with a central hyperchromatic nucleus and dense eosinophilic keratinized cytoplasm. Between the tumor nests, entrapped renal parenchyma in the form of a few cystically dilated and predominantly atrophic tubules and sclerosed glomeruli were seen. Perineural invasion was present. The renal sinus and perinephric fat were uninvolved. Foci of foamy histiocytic cell collections, lymphocytes, and plasma cells were in the surrounding renal parenchyma. On immunohistochemistry, the tumor cells were immunopositive for p40. Fine needle aspiration cytology of the liver lesion revealed atypical squamous cells in a necrotic background. The tumor was diagnosed as a renal SCC with focal areas of xanthogranulomatous pyelonephritis. The renohepatic fistula was formed by the contiguous malignant invasion of the adjacent liver.

The patient refused adjuvant therapy. His fever had resolved and his general well-being had improved at 2-month follow-up, but the patient died from acute myocardial infarction 6 months after surgery.

## DISCUSSION

Renal SCC is a rare high-grade neoplasm that is generally in an advanced stage at presentation, thus having a poor prognosis. The usual reported age of presentation of renal SCC is the fifth to the seventh decade, similar to the more common renal cell carcinoma (RCC), which has a better prognosis. The associated causative factors for SCC are recurrent urinary tract infections with chronic pyelonephritis, long-standing staghorn-type calculi, smoking, schistosomiasis, hormonal imbalance, analgesic abuse, and previous surgery for renal calculi.^4,5^ Most renal SCCs are associated with nephrolithiasis, but renal SCCs have no specific radiologic features.^1^

Because of the rarity of this malignancy, the literature is scarce. Renal SCC can have varied presentations such as diffusely enlarged nonfunctioning kidney with renal calculi, diffuse wall thickening with the absence of a distinct mass, hydronephrosis, and low echogenicity in the renal parenchyma or solid-cystic mass.^1,3,4^ On imaging, enhancing lesions with the exophytic or intraluminal components in the kidney can be a helpful feature for indicating the presence of renal SCC.^1^ The unpredictable imaging appearance often delays clinical/radiologic diagnosis. Histopathology generally clinches the final diagnosis. This situation is in contrast to RCC, in which patients may have classic symptoms of hematuria, flank mass, and flank pain, and typical radiologic features such as a solitary solid enhancing mass with decreased attenuation suggestive of necrosis are often present.

Renal SCCs originate more often from the renal pelvis than the renal parenchyma. Primary renal SCC needs to be differentiated from squamous differentiation in urothelial carcinoma and metastatic SCC.^2,5^ The closest differential diagnosis of SCC of renal origin is xanthogranulomatous pyelonephritis, an uncommon type of chronic pyelonephritis resulting from chronic obstruction, usually from renal stones and formation of an inflammatory mass destroying renal parenchyma and masquerading as malignancy.^6^ Xanthogranulomatous pyelonephritis can infiltrate adjacent organs, thus making it even more challenging to distinguish from malignancy.^6,7^

Renohepatic fistulas are rare and can result from trauma, infection (eg, tuberculosis, pyonephrosis, renal abscess rupture, liver abscess rupture into renal parenchyma), or, rarely, malignancy.^8^ Chung et al described a case of renal SCC leading to the development of a pyelo-colo-duodenal fistula.^8^ Chung et al also reported a pyelo-hepatic fistula that developed from the spread of infection from the kidney to the liver.^9^ A high index of suspicion on imaging and preoperative biopsy can aid in distinguishing between infection and malignancy.^10,11^ Our case clinically and radiologically appeared as xanthogranulomatous pyelonephritis; however, the histopathologic examination revealed an SCC of the kidney in a background of xanthogranulomatous pyelonephritis with metastases to the liver. Although primary renal SCC is rare, our case had contiguous liver invasion by renal SCC leading to a renohepatic fistula which, to our knowledge, has not been previously reported.^2,4,5,8-10,12-16^ This case highlights the highly variable presentations of renal SCC.

The primary treatment for renal SCC is nephrectomy, and the role of adjuvant chemotherapy or radiotherapy is uncertain.^3,13^ No standard guideline is available because of the rarity of this tumor. Our patient did not consent to adjuvant therapy. Further studies are essential to determine if chemotherapy or radiotherapy may improve survival, especially anti-epidermal growth factor receptor (EGFR) therapy in EGFR receptor–positive cases.^15^

## CONCLUSION

Renal SCC should be a differential diagnosis for any renal mass presenting as xanthogranulomatous pyelonephritis with renal calculi, long-term hydronephrosis, or thickened renal parenchyma. Extensive sampling of the specimen is essential to detect the presence of tiny unobvious foci of malignancy, especially in patients with xanthogranulomatous pyelonephritis or nephrolithiasis. Also, patients with nephrolithiasis should be regularly monitored for the development of malignancy.

## Notes

*This article meets the Accreditation Council for Graduate Medical Education and the American Board of Medical Specialties Maintenance of Certification competencies for Patient Care and Medical Knowledge.*
